# Supplementary material for: Differential Involvement of Autophagy and Apoptosis in Response to Chemoendocrine and Endocrine Therapy in Breast Cancer: JBCRG-07TR
Source: Int J Mol Sci. 2019 Feb 24;20(4):984. doi: 10.3390/ijms20040984 (PMC6412499; doi:10.3390/ijms20040984)
Supplement: Supplementary file 1 [file ijms-20-00984-s001.pdf]

Supplementary

Table S1. Baseline characteristics of patients in JBCRG-07 according to clinical response.

Characteristics		Number	
		Responder	Non-responder
Number of patients		26	12
Average age (range)		68.8 (57–81)	71.5 (61–82)
T	T1	1	0
	T2	22	11
	T3	3	1
N	N0	23	10
	N1	3	2
ER	+	26	12
	−	0	0
PgR	+	18	6
	−	8	6
HER2	+	6	2
	−	20	10
Histological grade	1	8	5
	2	18	7
	3	0	0

ER, estrogen receptor; PgR, progesterone receptor; HER2, human epidermal growth factor receptor 2.

Table S2. Baseline characteristics of patients in JFMC34-0601 according to clinical response.

Characteristics		Number	
		Responder	Non-responder
Number of patients		38	30
Average age (range)		65.9 (56–77)	65.1 (56–77)
T	T1	0	0
	T2	38	30
	T3	0	0
N	N0	26	24
	N1	12	6
ER	+	38	30
	−	0	0
PgR	+	34	28
	−	4	2
HER2	+	0	0
	−	38	30
Histological grade	1	4	4
	2	24	14
	3	2	7
	unknown	8	5

ER, estrogen receptor; PgR, progesterone receptor; HER2, human epidermal growth factor receptor 2.
